# Ligand-receptor-mediated attachment of lipid vesicles to a supported lipid bilayer

**DOI:** 10.1007/s00249-020-01441-0

**Published:** 2020-06-17

**Authors:** Vladimir P. Zhdanov

**Affiliations:** 1 Section of Biological Physics, Department of Physics, Chalmers University of Technology, Göteborg, Sweden; 2 Boreskov Institute of Catalysis, Russian Academy of Sciences, Novosibirsk, Russia

**Keywords:** Vesicles, Support, Multivalent ligand-receptor interaction

## Abstract

The interaction of exosomes (cell-secreted $\sim$100 nm-sized extracellular vesicles) or membrane-enveloped virions with cellular lipid membranes is often mediated by relatively weak ligand-receptor bonds. Interactions of this type can be studied using vesicles and observing their attachment to receptors located in a lipid bilayer formed at a solid surface. The contact region between a vesicle and the supported lipid bilayer and accordingly the number of ligand-receptor pairs there can be increased by deforming a vesicle. Herein, I (i) estimate theoretically the corresponding deformation energy assuming a disk-like or elongated shape of vesicles, (ii) present the equations allowing one to track such deformations by employing total internal reflection fluorescence microscopy and surface plasmon resonance, and (iii) briefly discuss some related experimental studies.

## Introduction

**Fig. 1:**
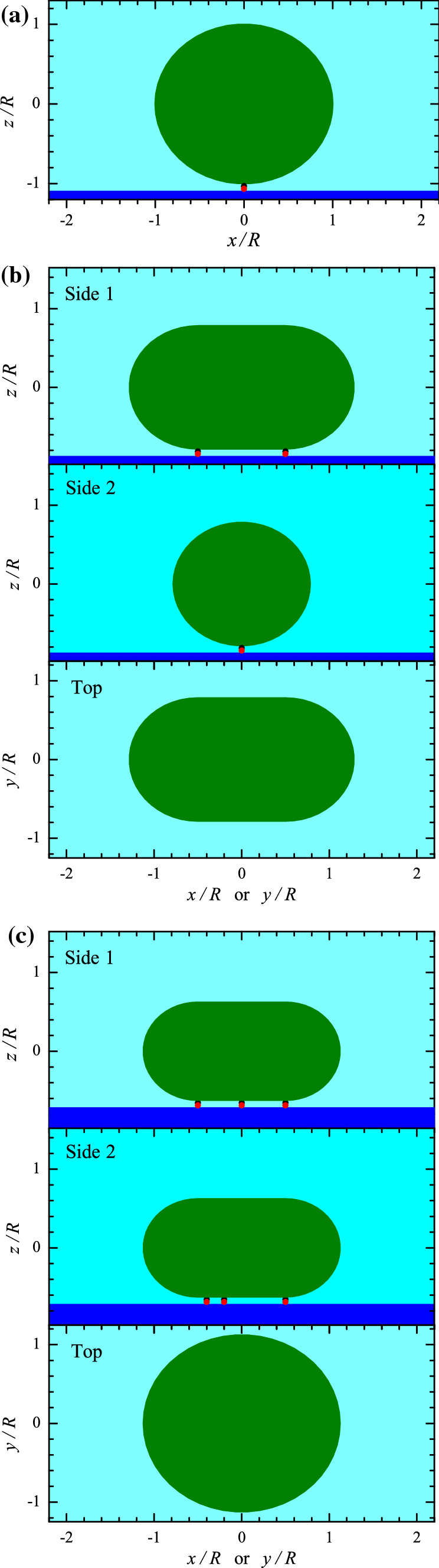
**a** Spherical, **b** elongated, and **c** disk-like vesicles attached to the surface by one, two, or three ligand-receptor pairs. Panels **b** and **c** show the side- and top-view projections

Lipid vesicles can often directly attach to solid surfaces and be intact or ruptured and form a supported lipid bilayer [SLB; see e.g. the articles by Cho et al. (2010), Mapar et al. (2018) and references therein]. Such SLBs can also be fabricated by using other approaches including e.g. the solvent-assisted lipid bilayer and bicelle methods (Jackman and Cho 2020). In applications, an SLB can contain receptors, and this platform allows one to study various aspects of the ligand-receptor-mediated interaction of vesicles, membrane-enveloped virions, virus-like particles, or lipid nanoparticles (of $\sim 100$ nm size) with lipid membranes as schematically shown in Fig. 1. In nature, such interactions between exosomes (cell-secreted $\sim$100 nm-sized extracellular vesicles) or membrane-enveloped virions and cellular lipid membranes are extremely important from various perspectives as reviewed by Kalluri and LeBleu (2020) and Mercer et al. (2010). The interaction of lipid nanoparticles with lipid membranes is also of high current interest from the perspective of the development of new drugs (Tibbitt et al. 2016; Zhdanov 2019).

If the attachment of a vesicle is mediated by one ligand-receptor pair, a vesicle can keep its shape, e.g., remain spherical (Fig. 1a). The formation of two or more ligand-receptor pairs may require appreciable vesicle deformation provided the distance between the pairs is comparable to or larger than the vesicle radius, *R* (Fig. 1b,c). If there are a few ($n>1$) or many ($n\gg 1$) ligand-receptor pairs, the gain in the energy due to the formation of additional ligand-receptor bonds (i.e., those formed after the formation of the first bond) is $-(n-1) I$, where $I>0$ is the bond energy, while the loss of energy is equal to the increase of the lipid-bilayer bending energy, $\Delta E_{\mathrm{b}}$ (provided the osmotic pressure is negligible). Thus, the formation of extra ligand-receptor pairs is favourable if

1 $$\begin{aligned} (n-1) I > \Delta E_{\mathrm{b}} . \end{aligned}$$

This simple condition neglecting the entropic factors shows that the shape of a vesicle is determined by the interplay between the ligand-receptor-bond formation and membrane bending.

Experimentally, the deformation of vesicles during attachment to solid surfaces in general and in the case of ligand-receptor mediation in particular can be tracked optically e.g. by using total internal reflection fluorescence microscopy [TIRFM; this technique is reviewed by Boukobza et al. (2001) and Olsson et al. (2015)], surface plasmon resonance [SPR; reviewed by Jung et al. (1998) and Rupert et al. (2016)], or localized surface plasmon resonance [LSPR; reviewed by Jackman et al. (2017)]. Vice versa, one can say that the vesicle deformation influences the TIRFM, SPR, and LSPR signals, and this effect can be important from the perspective of related applications of these techniques. Other techniques, e.g. crystal microbalance-dissipation [QCM-D; reviewed by Cho et al. (2010)], can be used here as well. In particular, some information about the deformation of vesicles directly attached to the support was already obtained by employing SPR [with emphasis on determining the size and concentration of sub-populations of extracellular vesicles (Rupert et al. 2016)], LSPR [with emphasis on the kinetics of vesicle attachment (Jackman et al. 2014), role of temperature (Oh et al. 2015) and divalent cations (Dacic et al. 2016), and deformation itself (Jackman et al. 2017)], and QCM-D [with emphasis on the role of osmotic pressure (Jackman et al. 2013)]. Despite these advances, one needs additional theoretical input for the use of these techniques.

Herein, I estimate $\Delta E_{\mathrm{b}}$ for $n\ge 2$ and present the equations allowing one to calculate the scale of the vesicle-deformation-related change of the TIRFM and SPR signals. The corresponding results are of intrinsic interest in the context of biophysics of multivalent interactions and also may potentially be useful in the context of development of sensors allowing the measurement of low concentrations of biological molecules via their attachment to a solid surface and playing the role of receptors by tracking their association with ligand-containing vesicles which can be employed as signal amplifiers.

**Fig. 2:**
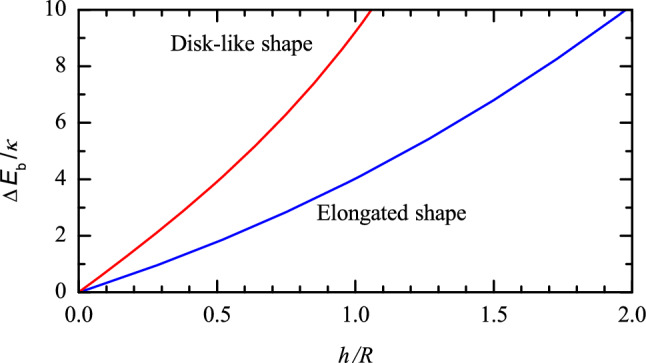
Increase of the vesicle bending energy as a function of *h*/*R* according to (12) and (16)

**Fig. 3:**
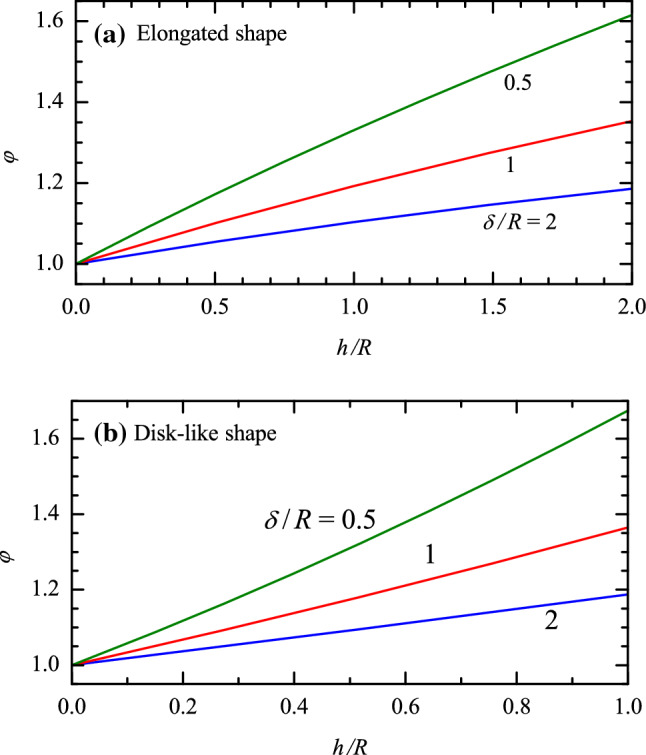
Factor describing the effect of the vesicle deformation on the TIRFM or SPR signal as a function of *h*/*R* at $\delta / R=0.5$, 1, and 2: **a** for elongated vesicles according to (26), and **b** for disk-like shape of vesicles according (27)

## Change of the bending energy

In the generic case without spontaneous curvature of a lipid bilayer, the vesicle bending energy can be represented as (Seifert 1997)

2 $$\begin{aligned} E_{\mathrm{b}} = \frac{\kappa }{2} \int (c_1+c_2)^2 ds, \end{aligned}$$

where $\kappa$ is the bending rigidity, $c_1=1/r_1$ and $c_2=1/r_2$ are the principal curvatures, $r_1$ and $r_2$ are the corresponding radii, and the integration is performed over the vesicle surface (*ds* is an element of the surface).

For spherically shaped vesicles with $c_1=c_2=1/R$, the bending energy is given by

3 $$\begin{aligned} E_{\mathrm{b}} = 8\pi \kappa . \end{aligned}$$

This conventional expression for the bending energy can be used to describe vesicles attached to the surface by one ligand-receptor pair (Fig. 1a).

In the case of a few ($n>1$) or many ($n\gg 1$) ligand-receptor pairs, the shape and exact value of the vesicle bending energy can be calculated via cumbersome numerical calculations (Seifert and Lipowsky 1990; Irajizad and Agrawal 2018). The results of such calculations are neither transparent nor convenient in applications. To get transparent results, I use simple physically reasonable approximations for the shape of deformed vesicles.

In particular, the vesicles attached to the surface via two ligand-receptor pairs ($n=2$) are represented as an elongated pellet (Fig. 1b) with hemispherical left and right parts (with radius *r*) and cylindrically shaped central part (with radius *r* and length *h*; this length can be identified with the distance between the two ligand-receptor pairs). In this approximation, the curvatures of the hemispherical parts are $c_1=c_2=1/r$, the curvatures of the cylindrical part are $c_1=0$ and $c_2=1/r$, and the vesicle bending energy is given by

4 $$\begin{aligned} E_{\mathrm{b}} = 8\pi \kappa + \pi \kappa h /r. \end{aligned}$$

The change of the bending energy is obtained by subtracting the initial energy (3), i.e.,

5 $$\begin{aligned} \Delta E_{\mathrm{b}} = \pi \kappa h /r . \end{aligned}$$

The two sizes used in (4) and (5) are not independent, because the lipid-bilayer areas of a vesicle before and after deformation, $4\pi R^2$ and $4\pi r^2 + 2\pi r h$, should be equal. This condition yields

6 $$\begin{aligned} R^2 = r^2 + r h/2 . \end{aligned}$$

According to this equation, *r* can be represented as a function of *h*,

7 $$\begin{aligned} r = (h^2/16 + R^2)^{1/2}-h/4, \end{aligned}$$

or *h* can be represented as a function of *r*,

8 $$\begin{aligned} h =2(R^2 - r^2)/r. \end{aligned}$$

In addition, it is convenient to introduce the maximum size of a deformed vesicle,

9 $$\begin{aligned} L=2r +h . \end{aligned}$$

This relation can be rewritten as $h=L-2r$. Substituting the latter relation into (6) yields

10 $$\begin{aligned} r=2R^2/L . \end{aligned}$$

Substituting this relation into (8), we obtain.

11 $$\begin{aligned} h=L-4R^2/L . \end{aligned}$$

Relations (7), (8), (10), and (11) allow us to rewrite (5) in terms of one of the sizes (*h*, *r*, or *L*) of a deformed vesicle,

12 $$\begin{aligned} \Delta E_{\mathrm{b}}= & {} \frac{\pi \kappa h }{(h^2/16 + R^2)^{1/2}-h/4}, \end{aligned}$$

13 $$\begin{aligned} \Delta E_{\mathrm{b}}= & {} 2\pi \kappa (R^2 - r^2)/r^2, \;\; \mathrm{or} \end{aligned}$$

14 $$\begin{aligned} \Delta E_{\mathrm{b}}= & {} \pi \kappa (L^2/2R^2 -2). \end{aligned}$$

The dependence of $\Delta E_{\mathrm{b}}$ on *h* is shown in Fig. 2.

The vesicles attached to the surface via three or more ligand-receptor pairs ($n\ge 3$) can be viewed as a rounded disk-like pellet with the peripheral radius *r* and cylindrically shaped central part of diameter *h* (Fig. 1c). Adopting this approximation, I employ the disk cross-section perpendicular to the surface and the maximum cross-section parallel to the surface to characterize the shape of the peripheral area. The corresponding curvatures are 1/*r* and $1/(r+h/2)$, respectively. With these curvatures, the vesicle bending energy is represented as

15 $$\begin{aligned} E_{\mathrm{b}}= \frac{\kappa }{2} \left( \frac{1}{r}+\frac{1}{r+h/2}\right) ^2 (4\pi r^2+ \pi ^2hr) . \end{aligned}$$

After subtracting the initial energy (3), the deformation-related change of the bending energy is given by

16 $$\begin{aligned} \Delta E_{\mathrm{b}}= \frac{\kappa }{2} \left( \frac{1}{r}+\frac{1}{r+h/2}\right) ^2(4\pi r^2+ \pi ^2hr) -8\pi \kappa . \end{aligned}$$

To relate *h* and *r*, I again take into account that the lipid-bilayer areas of a vesicle before and after deformation, $4\pi R^2$ and $4\pi r^2+ \pi ^2hr +\pi h^2/2$, should be equal, or

17 $$\begin{aligned} 4r^2+ \pi hr + h^2/2 = 4 R^2. \end{aligned}$$

This equation yields

18 $$\begin{aligned} r= & {} [(\pi ^2-8) h^2/64 +R^2]^{1/2}-\pi h/8, \;\; \mathrm{or} \end{aligned}$$

19 $$\begin{aligned} h= & {} [(\pi ^2 -8)r^2+8R^2]^{1/2}-\pi r . \end{aligned}$$

The maximum size of a deformed vesicle is defined by analogy with (9),

20 $$\begin{aligned} L=2r +h. \end{aligned}$$

Using (18), (19), or (20), the change of the bending energy given by (16) can be expressed as a function of *h*, *r*, or *L* (see e.g. Fig. 2).

## TIRFM, SPR, and vesicle deformation

In TIRFM or SPR experiments with vesicles immobilized at the interface, the signal measured is induced by the evanescent field. In particular, the signal can in general be described as [see, e.g., the articles by Boukobza et al. (2001) and Olsson et al. (2015) for TIRFM and by Jung et al. (1998) and Rupert et al. (2016) for SPR, respectively]

21 $$\begin{aligned} I =AJ_0 \int \exp (-z/\delta ) ds, \end{aligned}$$

where *A* is a constant proportional to the concentration of vesicles at the interface, $J_0$ is the intensity (square of the amplitude) of the incident light, $\delta$ is the decay length of the light intensity, *z* is the coordinate perpendicular to the interface, and the integration is performed over the vesicle surface.

For spherically-shaped vesicles (Fig. 1b; Eq. 3), expression (21) yields (Olsson et al. 2015; Rupert et al. 2016)

22 $$\begin{aligned} I(R) = 2\pi AJ_0 R\delta [1-\exp (-2R/\delta )]. \end{aligned}$$

If vesicles are represented as an elongated pellet (Fig. 1b; Eqs. 4–14), expression (21) results in

23 $$\begin{aligned} I(r,h)=2\pi AJ_0 r \{\delta [1-\exp (-2r/\delta )] +h \exp (-r/\delta ) I_0(r/\delta )\}, \end{aligned}$$

where

24 $$\begin{aligned} I_0(z)= \frac{1}{\pi }\int _0^{\pi } \exp (z \cos \phi ) d \phi , \end{aligned}$$

is the modified Bessel function of the first kind and order zero. Expression (23) can be rewritten as

25 $$\begin{aligned} I(r,h)=I(R) \varphi (r,h), \end{aligned}$$

where

26 $$\begin{aligned} \varphi (r,h) \equiv \frac{I(r,h)}{I(R)} = \frac{r \{\delta [1-\exp (-2r/\delta )] +h \exp (-r/\delta ) I_0(r/\delta )\}}{R \delta [1-\exp (-2R/\delta )]}, \end{aligned}$$

is the dimensionless factor ($\le 1$) describing the effect of the vesicle deformation on the TIRFM or SPR signal.

If vesicles are represented as a rounded disk-like pellet (Fig. 1c; Eqs. 15–20), expression (21) yields

27 $$\begin{aligned} \begin{array}{ll} I(r,h)= &{} \pi AJ_0 \{2r \delta [1-\exp (-2r/\delta )]\\ &{} +\pi h r \exp (-r/\delta ) I_0(r/\delta )\\ &{} + (h^2/4)[1+\exp (-2r/\delta )]\}. \end{array} \end{aligned}$$

This expression can also be represented as (25) with $\varphi (r,h) \equiv I(r,h)/I(R)$, where *I*(*r*, *h*) and *I*(*R*) are defined by (27) and (22).

## Discussion and conclusions

The results presented (Fig. 2) indicate that the vesicle bending energy becomes comparable with $2\kappa$ already for modest vesicle deformations, at $h/R \simeq 0.5$ and 0.25 for the elongated and disk-like shapes, respectively. Such deformations can be tracked by using TIRFM or SPR (Fig. 3). My analysis forms a basis for the corresponding experiments. The first part of the analysis (“Change of the bending energy”) can also be used for the interpretation of the data obtained by employing other techniques (e.g., QCM-D as briefly discussed below). In particular, it allows one to discuss the interplay between the bending energy and the free energy of the formation of the ligand-receptor pairs. This interplay is complex, and usually one set of measurements does not make it possible to accurately estimate all the desirable parameters. Some limitations on or relation between the parameters can, however, be obtained.

In the theoretical literature, one can often read that $$\kappa \simeq 25$$$$k_{\mathrm{B}}T$$ (Smith et al. 2004). The recent experiments with $$\sim 100$$ nm-sized vesicles indicate that $$\kappa $$ may be appreciably larger, up to $$\simeq 10^3$$$$k_{\mathrm{B}}T$$, even in conventional lipid bilayers [see the LSPR study by Jackman et al. (2017) and references therein]. For biomimetic lipid compositions containing e.g. cholesterol [reviewed by Cebecauer et al. (2018) and Enkavi et al. (2019)], $$\kappa $$ can be appreciably larger as well. Even if $$\kappa $$ is relatively small, $$\simeq 25$$$$k_{\mathrm{B}}T$$, the above-mentioned increase of the bending energy ($$\simeq 2\kappa \simeq 50$$$$k_{\mathrm{B}}T$$) is appreciable and can hardly be fully compensated by a few relatively weak bonds (smaller or about 5 kcal/mol or $$\simeq 8$$$$k_{\mathrm{B}}T$$) which are typical for the multivalent ligand-receptor interactions. With increasing vesicle size and/or length of ligand-receptor pairs, the number of ligand-receptor pairs located in the contact area can be large, and they can induce appreciable deformation of a vesicle.

To link the general conclusions above to real systems, one can look through a recent QCM-D study of attachment of biotinylated small (SUVs, $$\simeq 100$$ nm in diameter) and giant (GUVs, $$\simeq 15$$–20 $$\mu $$m in diameter) unilamellar DOPC vesicles to a biotinylated SLB functionalized with streptavidin (Di Iorio et al. 2020). One of the advantages of this choice of species is that the biotin-streptavidin interaction has long been used for biomolecule immobilization, and its strength is well established [$$K_{\mathrm{d}}\simeq 10^{-14}$$ M (Di Iorio et al. 2020) or $$\Delta G =18.3$$ kcal/mol (Weber et al. 1992)]. In the experiment, the fraction of biotinylated lipids, *f*, in an SLB was varied from 0.002 to 0.02, whereas in vesicles it was from 0.001 to 0.02. Analyzing the QCM-D data obtained for SUVs, the authors have concluded that the deformation of SUVs was modest (with the SUV surface-contact area $$\le 10\%$$) provided $$f\le 0.006$$ (in an SUV), and under such conditions the number of biotins (on the SUV side) involved in the contact was $$\le 130$$. The formation of ligand-receptor pairs in the SUV-SLB contact area is accompanied by the loss of entropy of ligands and receptors. In the context under consideration, this loss per pair can be estimated as $$\simeq -2k_{B}T\ln (f)$$ or $$\simeq 6$$ kcal/mol (provided $$f=0.006$$). The bond-formation gain in the free energy can be estimated by subtracting this value from $$\Delta G$$, i.e., about 18–6=12 kcal/mol per pair. For 130 pairs, this gain is $$\simeq 1500$$ kcal/mole or $$\simeq 2500$$$$k_{\mathrm{B}}T$$. Identifying the latter value with $$2\kappa $$ (as suggested in the first paragraph of this section), I obtain $$\kappa \simeq 1200$$$$k_{\mathrm{B}}T$$. Thus, this analysis also indicates that $$\kappa $$ can be much larger than 25 $$k_{\mathrm{B}}T$$. Concerning GUVs, their deformation of attached was observed explicitly by using fluorescence microscopy (Di Iorio et al. 2020), but the discussion of the corresponding results is beyond our goals.

In another study, the deformation of SUVs ($\simeq 100$ nm in diameter and of complex composition) adhered to poly-L-lysine coated glass was studied by using AFM (Vorselen et al. 2017). Referring to the conventional bending rigidity, $\kappa \simeq 10$-50 $k_{\mathrm{B}}T$, and accepting this scale of $\kappa$ in the calculations, the authors have concluded that the bilayer bending alone cannot account for the high stiffness observed experimentally and refer to relatively high osmotic pressure (in their analysis, this pressure was used as a fitting parameter and estimated to be $\simeq 0.15$ MPa) to explain their observations. The discussion above shows that one cannot exclude that the bending rigidity might in reality be much higher than 10–50 $k_{\mathrm{B}}T$.

Finally, I can add that, although my work was motivated by referring to the ligand-receptor-mediated interaction of vesicles with an SLB, the results reported can be used in a more general context. In my analysis, the role of an SLB is in fact reduced to prevention of a direct contact of vesicles with a support, and accordingly the bilayer can be replaced by any other layer preventing such a contact (Kim et al. 2017). If a support itself does not interact with vesicles, it can be directly (without an SLB) functionalized by receptors (Oliverio et al. 2017; Liu et al. 2020). For example, a support can be isotropic or anisotropic and contain parallel protrusions so that it can induce the formation of elongated vesicles (Fig. 1b) after their attachment. In such situations, all the results presented can be valid. Some of the results may be valid even if vesicles interact not only with receptors but also directly with a functionalized support.
